# Comprehensive Genomic Analysis and Expression Profiling of the C2H2-Type Zinc Finger Protein Family Under Abiotic Stresses in Watermelon

**DOI:** 10.3390/genes17050504

**Published:** 2026-04-24

**Authors:** Siyu Zhang, Yanuan Zhu, Hailiang Yu, Shihui Yao, Tao Xiao, Yongchao Yang, Chao Li, Hao Li, Jianxiang Ma, Yong Zhang, Xian Zhang, Chunhua Wei, Zhongyuan Wang

**Affiliations:** 1 State Key Laboratory of Crop Stress Biology in Arid Areas, College of Horticulture, Northwest A&F University, Yangling 121100, China; zhangsiyu@nwafu.edu.cn (S.Z.); zhuyanuan@nwafu.edu.cn (Y.Z.); 15603601090@163.com (H.Y.); ysh2023@nwafu.edu.cn (S.Y.); 2022010569@nwafu.edu.cn (T.X.); yuanyilihao123@163.com (H.L.); majianxiang@126.com (J.M.); zhangyong123@nwafu.edu.cn (Y.Z.); zhangxian@nwafu.edu.cn (X.Z.); 2 College of Biological and Agricultural Sciences, Honghe University, Mengzi 661199, China; yangyongchaos@foxmail.com; 3 Xinjiang Uygur Autonomous Region Research and Development Center for Facility Agriculture and Specialty Agriculture, Turpan 838200, China; lclxblc@126.com

**Keywords:** C2H2-type zinc finger protein, watermelon, whole genome identification, abiotic stresses, expression analysis

## Abstract

Background: C2H2 zinc finger proteins (C2H2-ZFPs) are one of the largest transcription factor families in plants and play vital roles in plant organ development and patterning, seed germination, and fruit ripening, as well as responses to biotic and abiotic stresses. Although widely studied in many species, the genome-wide characterization of the *C2H2-ZFP* family in watermelon (*Citrullus lanatus*) remains lacking. Methods: In this study, we identified 96 *ClZFP* genes in the watermelon genome and analyzed their chromosomal positions, gene structures, conserved motifs, and expression profiles. A tissue-specific expression analysis of 12 representative *ClZFP* genes revealed diverse and organ-preferential expression profiles, indicating functional differentiation during development. Results: Under abiotic stress treatments, four genes were significantly downregulated under drought, while one gene was strongly induced; six genes were inhibited and three genes were activated under low temperature; and most tested genes were upregulated at 72 h under salt stress, with one gene continuously induced throughout the treatment period. Key *ClZFP* members such as *ClZFP36* and *ClZFP72* showed specific and strong induction under drought and salt stress, respectively. Conclusions: These results indicate that *ClZFPs* may be involved in the tolerance of watermelon to various abiotic stresses. This study not only clarifies the evolutionary and expression characteristics of the *ClZFP* family in watermelon but also provides candidate genes for the genetic improvement of stress tolerance in cucurbit crops.

## 1. Introduction

Zinc finger transcription factors (ZF-TFs) constitute one of the most extensive transcription factor families within eukaryotes, characterized primarily by the presence of zinc finger domains [1]. The canonical zinc finger (ZF) motif is defined by the consensus sequence CX_2-4_CX_3_FX_5_LX_2_HX_3-5_H, which harbors two cysteine residues and two histidine residues. These amino acid residues coordinate a single zinc ion in a tetrahedral configuration, folding into a stable, compact domain that is specialized for nucleic acid binding [2]. Variations in the combination of these coordinating residues give rise to a diverse array of zinc finger architectures, including C2H2, C2HC, C2C2, hybrid C2HC/C2C2, and tandem C2C2 dimers [3]. Among these variants, the Cys_2_His_2_ (C_2_H_2_) type zinc finger domain was first identified in the *Xenopus oocytes* transcription factor TFIIIA, and it belongs to one of the largest subfamilies of zinc finger architectures [4]. Structurally, the C2H2 zinc finger domain is a polypeptide segment of approximately 22 amino acids, featuring the core consensus sequence CX_2-4_FX_5_LX_2_HX_3-5_H. In this motif, two cysteines and two histidines form a tetrahedral coordination complex with a zinc ion, which drives the folding of the polypeptide into a characteristic finger-like conformation composed of a β-hairpin and an α-helix [5]. Since the first plant-derived C2H2-type zinc finger protein gene, EPF1, was cloned from *Petunia hybrida*, the C2H2 protein family has been the subject of extensive characterization across a broad range of plant species [6]. Through systematic bioinformatics analyses, numerous members of the C2H2-type zinc finger protein (C2H2-ZFP) family have been identified in model and crop plants alike, including Arabidopsis, rice, wheat, citrus, apple, cucumber, and rapeseed [7,8,9,10,11,12,13].

Mounting research evidence has established that C2H2-ZFPs serve as key regulators of multiple biological processes in plants, encompassing abiotic stress tolerance and biotic stress resistance, as well as various aspects of growth and development. In *Arabidopsis thaliana*, *ZAT18*—a nuclear-localized C2H2-ZFP protein—acts as a positive regulator and plays a pivotal role in mediating plant adaptive responses to drought stress [14]. In tomato, *SlZF3* enhances salt tolerance by promoting the accumulation of ascorbic acid (ASA), which further illustrates the functional versatility of C2H2-type transcription factors in plant salt stress responses [15]. C2H2 zinc finger proteins are also critical mediators of plant responses to low-temperature stress; for example, *AtZAT6* activates the CBF (C-repeat binding factor) pathway, which is essential for melatonin-mediated freezing tolerance in *A. thaliana*, while *OsCTZFP8* confers enhanced cold tolerance in rice [16,17].

Beyond abiotic stress adaptation, C2H2-ZFPs also play indispensable roles in shaping plant physiological and molecular defense systems against pathogen infections. In pepper, *CaZNF830* acts as a positive regulator of immunity against *Ralstonia solanacearum* inoculation (RSI) [18]. In grapevine (*Vitis vinifera*), *VvZFP11* is strongly induced by the immune-related hormones salicylic acid (SA) and methyl jasmonate (MeJA), and it also rapidly responds to *Erysiphe necator* (powdery mildew) infection [19]. Similar regulatory functions in biotic stress resistance have been documented in other plants. For example, the overexpression of *StZFP2* enhances potato resistance to *Phytophthora infestans*-caused late blight, whereas the silencing of *NbCZF1* weakens resistance against *Phytophthora nicotianae* in *Nicotiana benthamiana* [20,21].

In addition to their roles in enhancing plant tolerance to adverse environmental and biotic stresses, C2H2-ZFPs also exert crucial regulatory functions in plant growth, development, and secondary metabolic processes. In *A. thaliana*, the protein MAZ1 mediates primexine deposition and sporopollenin assembly, which are critical for pollen wall formation and fertility [22]. Similarly, the rice C2H2 transcription factor *STAMENLESS 1* (*SL1*) is indispensable for normal floral organ development [23]. Notably, C2H2 transcription factors in these developmental processes frequently function by coordinating with plant hormone signaling pathways. For instance, *A. thaliana ZFP3* and its homologous subfamily regulate light and abscisic acid (ABA) signaling during seed germination and early seedling growth [24]. In tobacco, the C2H2 transcription factor *NbGIS* promotes glandular trichome initiation by integrating gibberellin (GA) signaling [25]. Taken together, the mounting evidence demonstrates that C2H2-ZFPs serve as pivotal regulators in plants, mediating adaptive responses to abiotic and biotic stresses as well as governing key growth and developmental processes, often via synergistic interactions with phytohormone signaling pathways.

Watermelon, a member of the *Cucurbitaceae* family, is not only an important agricultural and economic crop but also an annual horticultural crop. It contributes significantly to both the fresh produce market and the processed food industry, and it plays a vital role in increasing farmers’ income and enriching dietary diversity. Preliminary studies have shed light on the involvement of C2H2 transcription factors in specific physiological processes of the watermelon; for instance, in trials conducted to investigate plant adaptive responses to low-nitrogen availability, transcriptome sequencing data demonstrated that a subset of C2H2 transcription factor family members participate in mediating nitrogen utilization and homeostasis in the foliar and root tissues of watermelon [26]. Another notable example is the gene *ClWIP1*, which encodes a C2H2-ZFP transcription factor and serves as a core regulatory gene mediating sex determination in watermelon; a targeted knockout of this gene yields exclusively gynoecious watermelon lines [27]. Although functional studies have been conducted on several C2H2 genes in watermelon, comprehensive genome-wide identification, structural characterization, and stress-responsive expression analysis of the entire C2H2-ZFP gene family in watermelon have not been systematically performed. Therefore, the objective of this study is to conduct a genome-wide characterization of the C2H2-ZFP gene family in watermelon and clarify the gene expression profiles in response to stress conditions. The results of this study not only provide target genes for further functional studies on the C2H2 family but also lay a foundation for investigating the molecular regulatory mechanisms of watermelon C2H2 transcription factors in response to abiotic stresses.

## 2. Materials and Methods

### 2.1. Plant Materials and Treatments

In this study, the watermelon cultivar ‘M08’ was employed as the experimental material, which was provided by Cucurbit Germplasm Innovation and Genetic Improvement Laboratory, College of Horticulture, Northwest A&F University. Watermelon seedlings were grown in a growth chamber programmed at a day/night temperature regime of 28/18 °C, a 12 h photoperiod, and a photosynthetic photon flux density (PPFD) of 600 μmol·m^−2^·s^−1^. When watermelon seedlings reached the four-leaf stage, they were exposed to low-temperature, salt, and drought stresses, respectively. For low-temperature stress, seedlings were maintained at 4 °C. Leaf samples were harvested at 3, 6, 12, 24, and 36 h after treatment. Regarding drought stress, seedlings were fully irrigated prior to the initiation of water withholding, and leaf samples were collected at 0, 2, 4, 6, 8, and 10 d after the last watering. As for salt stress, seedlings were treated with 50 mL of 250 mM NaCl solution, and leaf samples were collected at 0, 3, 6, 12, 24, 36, 48, and 72 h after salt application. All leaf samples were collected in triplicate biological replicates, immediately frozen in liquid nitrogen, and stored at −80 °C for subsequent RNA extraction. Samples collected at 0 h or 0 d were used as the control. To investigate tissue-specific expression patterns, samples of leaf, root, stem, tendril, female flower, and male flower were collected. All tissue samples were prepared in triplicate, snap-frozen in liquid nitrogen, and stored at −80 °C until further processing.

### 2.2. Identification and Sequence Analysis of ClZFP Family Members

Genome-wide identification of C2H2-ZFP (*ZFP*) genes was performed based on the released watermelon genome (Cucurbit Genomics Database (http://cucurbitgenomics.org/v2)). The latest hidden Markov models (HMMER3.1 E-value = 0.01) corresponding to the C2H2-type domains, PF00096 and PF13912, were retrieved from the Pfam database (http://pfam.xfam.org/, version 33.1) and used as query models for the analysis. Putative *ClZFP* genes were further validated for identified candidate homologs via SMART (http://smart.embl-heidelberg.de). The physicochemical properties of *ClZFP* gene family members, including molecular weights (MWs), isoelectric points (pIs), grand average of hydropathicity (GRAVY) and the number of amino acids, were analyzed using online tools available on the ExPASy Proteomics Server (https://www.expasy.org/). The subcellular localization of these ClZFP proteins was predicted using the Euk-mPLoc 2.0 server (http://www.csbio.sjtu.edu.cn/bioinf/euk-multi-2/). All the related obtained information of these *ClZFP* genes is listed in the Supplemental Table S1.

### 2.3. Chromosome Distribution and Phylogenetic and Collinearity Analysis

The genomic location information of *ClZFP* genes was acquired from the Cucurbit Genomic Database. All *ClZFP* family members were physically mapped to the corresponding watermelon chromosomes, and their chromosomal distribution patterns were visualized by TBtools (https://github.com/CJ-Chen/TBtools). Based on the existing research on the ZFP family genes in *A. thaliana* the protein sequences of ZFPs in *A. thaliana* were downloaded from the TAIR database (http://www.arabidopsis.org/). The cucumber genome sequences were acquired from the Cucurbit Genomics Database (CuGenDB) [12]. These sequences were compared with the ZFP protein sequences of watermelon using CLUSTALW. An evolutionary tree was constructed using MEGA X software. The maximum likelihood (ML) method was employed, and the parameters were verified by Bootstrap with a 1000-iterations bootstrap test. Furthermore, collinearity analysis was conducted. The syntenic blocks and duplicated gene pairs between Arabidopsis and watermelon were identified using TBtools and displayed using a Circos plot.

### 2.4. Gene Structure and Motif Analysis

To characterize the gene structures of the *ClZFP* family genes, the online tool GSDS 2.0 (http://gsds.cbi.pku.edu.cn/) was employed for the analysis of gene structures of ZFP family members. The amino acid sequences of *ClZFP* family members were retrieved from the Cucurbitaceae Database. Motif prediction was conducted using the MEME Server v4.12.0 (http://meme-suite.org/tools/meme), in which the parameter of a motif width larger than 10 and less than 50 was used. The exon–intron structures of *ClZFP* genes were analyzed and plotted with GSDS2.0 software (Gene Structure Display Server 2.0 (gao-lab.org).

### 2.5. RNA Isolation and Gene Expression Analysis

Plant RNA Kit (GENENODE, Beijing, China) was used for the extraction of total RNA from samples, strictly following the manufacturer’s instructions. The quality and purity of total RNA was determined by measuring concentration and purity using a spectrophotometer (NanoDrop, Wilmington, DE, USA) and RNA integrity was further verified by agarose gel electrophoresis. Subsequently, first-strand cDNA was synthesized utilizing the FastKing RT Kit with gDNase (TIANGEN, Beijing, China). The CDS sequences of *ClZFP* genes were retrieved from the Cucurbit Genomics Database (watermelon genome database, http://cucurbitgenomics.org/). Twelve representative *ClZFP* genes were randomly chosen from clades containing more family members for expression pattern analysis. Gene-specific primers for members of the watermelon *ClZFP* gene family were designed with Primer 7.0, and the detailed primer sequences are listed in Table S2. Quantitative real-time PCR (qRT-PCR) was carried out with the SYBR Green PCR Master Mix (Applied Biosystems, Foster City, CA, USA) on an IQ5 Multicolor Real-Time PCR System (Bio-Rad). Reverse-transcribed cDNA served as the amplification template, and watermelon *ClActin* was used as the internal reference gene [28]. Relative quantification was calculated according to the 2^−ΔΔCT^ method described by Livak and Schmittgen, with three biological replicates performed for each sample [29]. Student’s *t*-test was used to determine significant differences at the same period between control group and treatment group. Heat maps were generated using TBtools software, statistical significance analysis was performed using IBM SPSS Statistics (version 31.0.0), and all experimental plots were constructed using GraphPad Prism (Prism 11) software.

## 3. Results

### 3.1. Identification and Characterization of ClZFP Family Members

A total of 96 members of the *ClZFP* gene family were identified in the watermelon genome and designated as *ClZFP1* to *ClZFP96* on the basis of their chromosomal positions. The amino acid number, molecular weight, isoelectric point (pI), grand average of hydropathicity (GRAVY), and subcellular localization of these proteins were analyzed (Table 1). The results showed that the amino acid lengths of the ClZFP family members ranged from 131 aa (ClZFP62) to 1561 aa (ClZFP25), and their molecular weights (MWs) varied from 14.60 kDa (ClZFP54) to 174.87 kDa (ClZFP25). In addition, the maximum and minimum isoelectric points (pIs) of the ClZFP proteins were 9.94 (ClZFP73) and 4.75 (ClZFP20), respectively. The GRAVY values of all the ClZFP proteins were below zero, indicating that these proteins were predicted to be hydrophilic. Based on the instability index values (>40), most of the ClZFP proteins were predicted to be unstable, with the exception of ClZFP23, ClZFP60 and ClZFP82. The aliphatic index of the ClZFP proteins ranged from 42.86 to 85.2, indicating relatively low thermostability. The subcellular localization prediction showed that most of the ClZFP proteins were putatively localized in the nucleus. Additionally, ClZFP7, ClZFP10, ClZFP26, ClZFP31, ClZFP40, ClZFP47, ClZFP56, ClZFP59, ClZFP60, and ClZFP95 are also predicted to localize to the cytoplasm.

### 3.2. Chromosomal Localization and Phylogenetic and Gene Duplication Analysis of ClZFP Family Members

**Figure 1:**
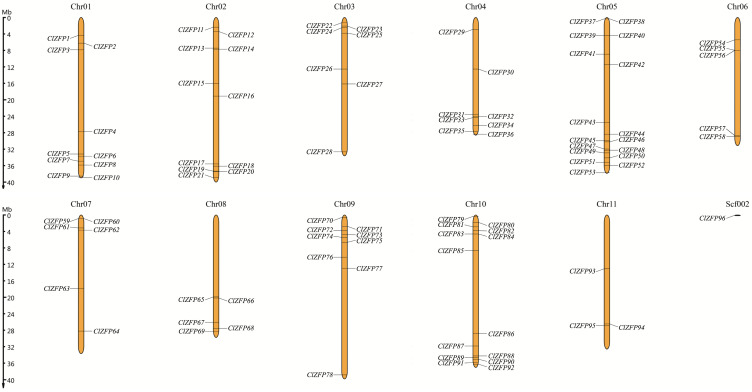
Distribution of *ClZFP* genes on watermelon chromosomes. The left scale represents the length of watermelon chromosomes with a unit of Megabase pairs (Mb).

The 96 *C2H2-ZFP* family members in watermelon were unevenly distributed across all 11 chromosomes, suggesting distinct expansion patterns among the chromosomal regions (Figure 1). Chromosome 10 harbored the largest number of ZFP genes (14 members), while chromosome 11 contained the fewest (only 3 genes). The ZFP gene Cla97C00G000030 (ClZFP96) was not assigned to any assembled chromosome.

**Figure 2:**
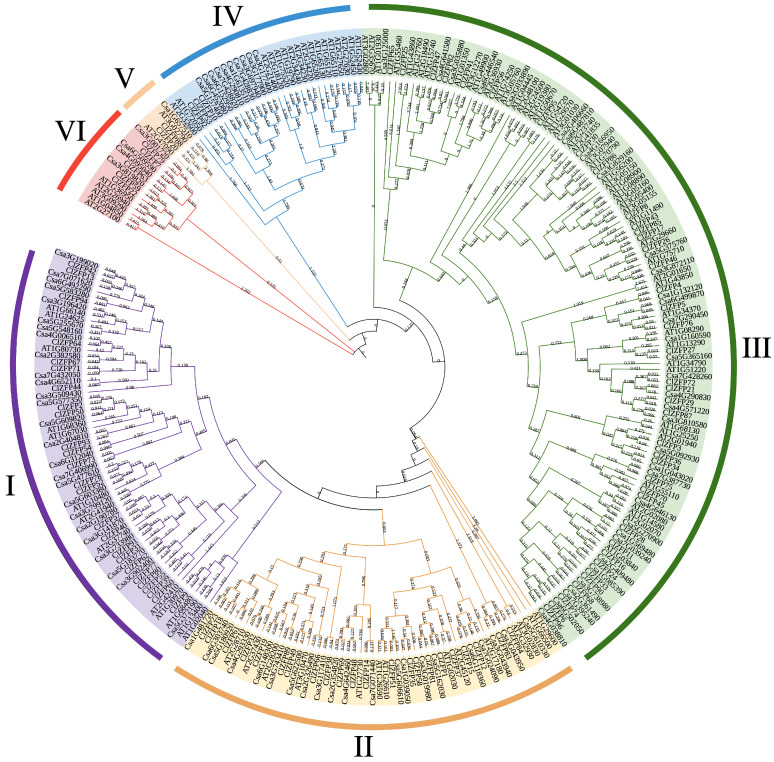
Interspecific phylogenetic tree of ClZFP proteins from watermelon, cucumber and *Arabidopsis*. The phylogenetic tree was constructed using the maximum likelihood (ML) method with partial deletion of 1000 bootstraps and a WAG model by MEGA X.

To further elucidate the evolution of the *ClZFP* gene family, a phylogenetic tree was constructed using the identified o align protein sequences from *A. thaliana*, cucumber (*Cucumis sativus*), and watermelon, and it was constructed using MEGA X (Figure 2). These ClZFP proteins were clustered into six clades. Clade I comprised 25 ClZFP members, Clade II contained 22 members, and Clade III included 39 members, representing the largest clade. Clade IV included three members, Clade V was the smallest clade with only two members, and Clade VI consisted of five members.

**Figure 3:**
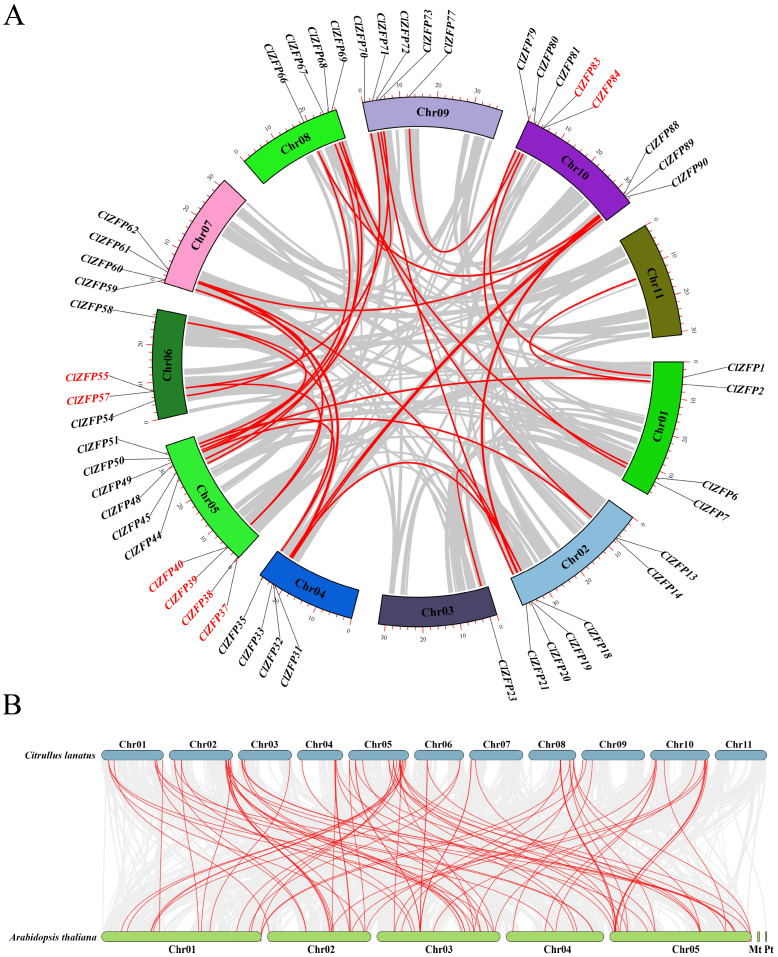
Gene duplication, and collinearity analyses of *ClZFP* genes. (**A**) Genome chromosomal localization and duplicated gene pairs of *ClZFP* genes in watermelon. The tandemly duplicated genes are indicated in red and the segmentally duplicated genes are indicated in black. (**B**) The syntenic relationship of *ClZFP* genes between watermelon and *A. thaliana*. The red curved lines represent orthologous gene pairs between watermelon and *A. thaliana*.

A gene duplication analysis revealed that 8 members were tandemly duplicated genes, while 42 members were segmentally duplicated genes (Figure 3A). To explore the evolutionary synteny of the *ClZFP* gene family between watermelon and *Arabidopsis*, a genome-wide collinearity analysis was conducted, and the syntenic blocks were visualized via a Circos plot (Figure 3B) with red lines marking the conserved genomic regions. A total of 59 homologous *ZFP* genes were identified in watermelon and *A. thaliana* (Table S3). Extensive many-to-many syntenic connections were identified across their nuclear genomes, with watermelon Chr01/02/05/10 and *Arabidopsis* Chr01/05 showing the densest links, while no synteny was detected between the watermelon nuclear genome and *Arabidopsis* organellar genomes (Mt/Pt). These *Arabidopsis ZFP* genes usually matched multiple homologous ZFP genes in watermelon; for instance, AT2G45120 was homologous to ClZFP1, ClZFP37, and ClZFP81, while AT1G80730 was homologous to ClZFP 44, ClZFP 67 and ClZFP71.

### 3.3. Gene Structures and Conserved Motif Analysis of ClZFP Family Members

**Figure 4:**
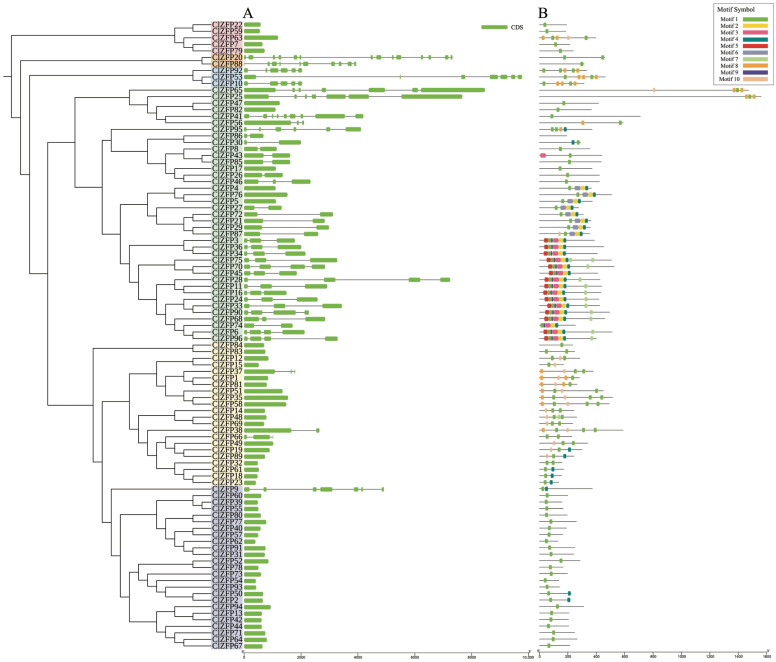
Gene structures and conserved motif analysis of *ClZFP*s in watermelon. (**A**) Exon–intron structures of *ClZFP* genes. The green boxes indicate the exons and the black lines indicate the introns. The bottom scale represents base pairs (bp). (**B**) The conserved motifs within the ClZFP proteins. Motifs 1 to 10 were indicated with different colors, and their consensus sequences are listed: motif 1: CEICGKGFQSDQALGGHRRSH; motif 2: KKWKCEKCSKKYAVQSDWKAHSKTCGTREYK; motif 3: KEVKKKVYVCPEPSCVHHDSRALGDLTGIKKHFCRKHGE; motif 4: DCGTLFSRGDSLIGHRAFCDA; motif 5: KRNLPGRPDPDAEVIALSPKTLLATNRFV; motif 6: MLRLPCYCCAEGCRNNNIDHPRSKPLKDFRTLQTHYKRKHG; motif 7: SPHMSATALLQKAAQMGATAS; motif 8: CKLCKKSFNGRALGGHMRSH; motif 9: NLPWKLRQRTS; motif 10: EEEEDLANCLIMLSRG.

The exon–intron structures of the *ClZFP* genes were analyzed and plotted with TBtools. As shown in Figure 4A, the exon numbers of these family members varied from 1 to 14, indicating high structural diversity within the family. Members with only one exon were the most abundant, totaling 54. *ClZFP20* contained the highest number of exons (14), followed by *ClZFP88* (10) and *ClZFP41* (9), which clustered in Clade V. To further explore functional conservation and divergence, ten conserved motifs were identified from 96 C2H2-type zinc finger proteins (Figure 4B). Among them, motifs 1, 2, and 3 represent the typical conserved domains of the C2H2-type zinc finger proteins. Motif 1 harbors a plant-specific conserved sequence (‘QALGGH’), which is present in the proteins of 95 family members, with ClZFP86 being the only exception. Notably, 35 members possess only motif 1 as their sole conserved motif. Most of these families are clustered in Clade I, implying a conserved core function and potentially reduced functional complexity. In addition, 11 family members harbor 7 motifs, the maximum number detected in the family, and 3 structurally analogous members contain 6 motifs. All of these members with a high motif complement are clustered within Clade III, hinting at potential functional specialization within this evolutionary lineage.

### 3.4. Expression Patterns of ClZFP Genes in Different Watermelon Tissues

**Figure 5:**
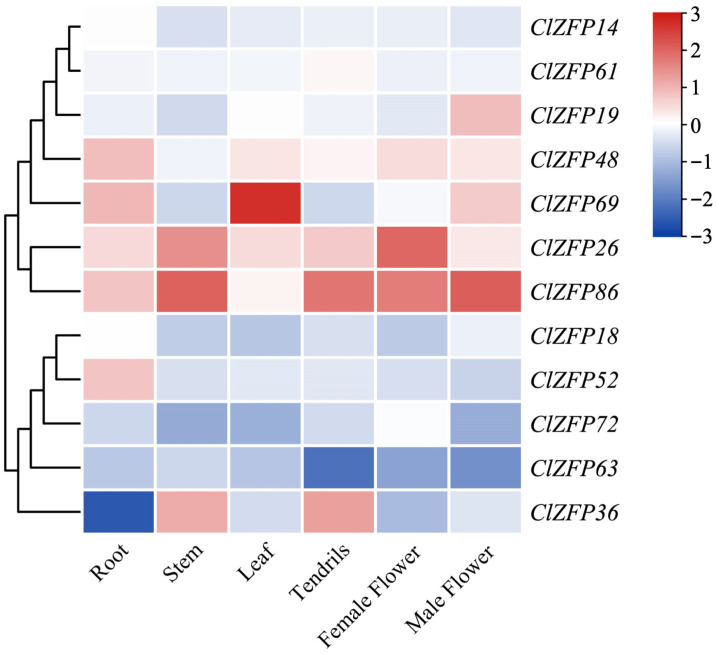
Expression analysis of *ClZFP* genes in different watermelon tissues. The color scale from blue to red represents lower to higher relative expression levels.

To explore the potential functionsof the *ClZFP* genes in watermelon, their expression patterns were analyzed among six distinct major tissues, including root, stem, leaf, tendril, female flower and male flower. As shown in Figure 5, these 12 *ClZFP* genes displayed diverse and tissue-specific expression profiles, suggesting functional differentiation among the family members. *ClZFP26* and *ClZFP86* showed constitutively high expression across all the tested tissues, indicating their potential broad roles in plant growth and development. Among them, *ClZFP86* exhibited relatively high expression in stems, tendrils, female flowers and male flowers. Similarly, *ClZFP26* showed the highest expression levels in stems and female flowers. Among the selected members of the *ClZFP* gene family, *ClZFP86* displayed the highest expression level in stem tissue, while *ClZFP69* showed extremely significantly high expression in leaf tissue; its expression level was much higher than in other tissues such as stems, tendrils and floral organs. Conversely, *ClZFP72* and *ClZFP63* exhibited low expression levels in the vast majority of the tissues. Specifically, *ClZFP63* maintained low expression in roots, stems, leaves and flowers, with its expression level reaching a minimum in tendrils. *ClZFP36* showed extremely significant negative expression in root tissue, which represented the lowest expression level of this gene among the six examined tissues.

### 3.5. Responses of ClZFP Genes to Drought, Low-Temperature and NaCl Treatments

**Figure 6:**
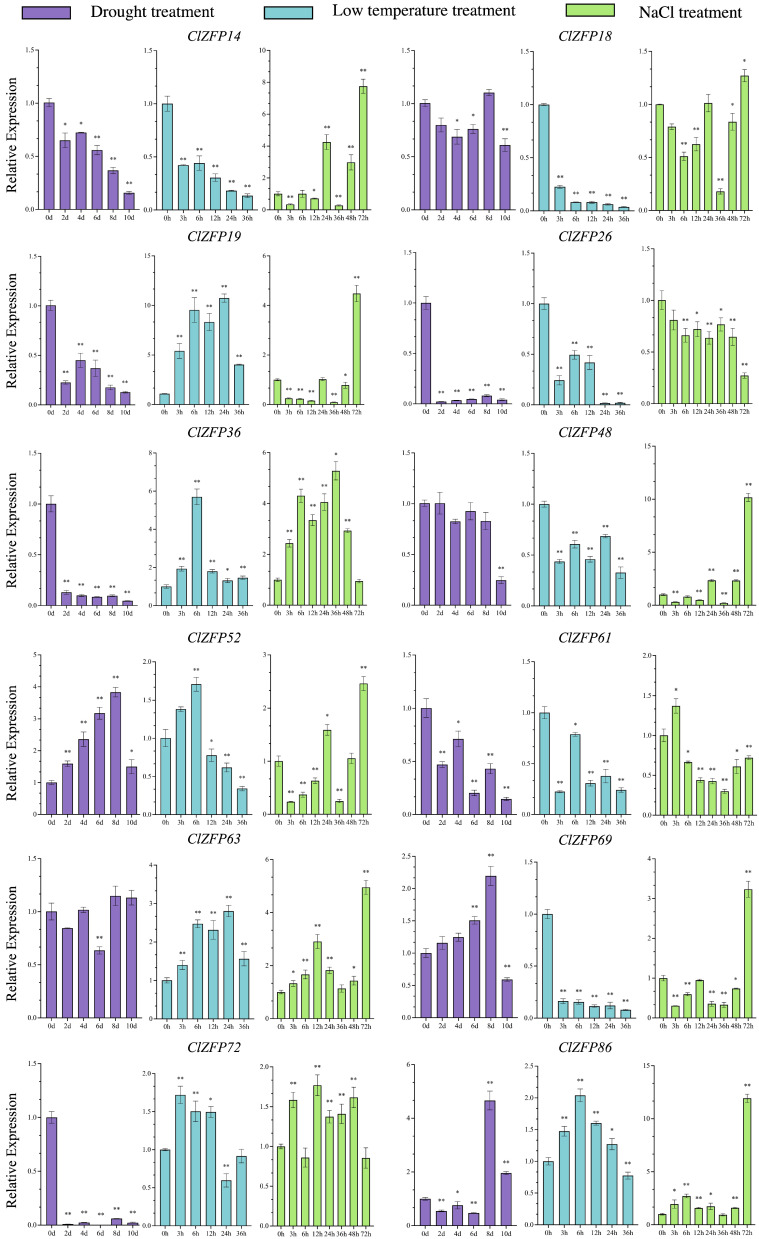
Expression analysis of the *ClZFP* genes under drought, low-temperature and NaCl treatments. Student’s *t*-test was used to determine significant differences at the same period between control group and treatment group. 0 h/0 d means the control without any treatment. Significance level: * *p* < 0.05. ** *p* < 0.01.

The expression patterns of the 12 *CIZFP* genes in response to drought stress, low-temperature treatment, and NaCl stress were characterized by quantitative real-time polymerase chain reaction (qRT-PCR) analysis (Figure 6). Across all the treatment conditions, the *CIZFP* genes displayed distinct stress-specific and time-dependent expression profiles.

Under drought conditions, most of the selected members of the *CIZFP* gene family exhibited a decreasing trend in their relative expression levels compared with the control as the treatment time progressed. Notably, *ClZFP19*, *ClZFP26*, *ClZFP36*, and *ClZFP72* were significantly repressed at all time points, suggesting a potential negative regulatory role or reduced activity under water-deficit conditions. In contrast, ClZFP52 was strongly and significantly induced by drought, indicating a positive regulatory function in the drought response. Furthermore, *CIZFP48* displayed a weak response to drought stress, with only a slight reduction in expression level observed at 10 days post-treatment.

In response to the low-temperature treatment, the selected members of the *CIZFP* gene family exhibited two distinct expression patterns. *ClZFP14*, *ClZFP18*, *ClZFP26*, *ClZFP48*, *ClZFP61*, and *ClZFP69* were significantly downregulated at all time points. In contrast, *CIZFP19*, *CIZFP36*, and *CIZFP63* exhibited the opposite expression pattern, with their expression levels being significantly upregulated compared with the control under low temperature, suggesting a role in cold tolerance. Furthermore, *CIZFP52*, *CIZFP72*, and *CIZFP86* displayed unique biphasic expression patterns. Specifically, *CIZFP52* was upregulated within the first 6 h of treatment, but it subsequently exhibited significant downregulation from 12 h post-treatment onward relative to the control. Similarly, *CIZFP72* was extremely significantly upregulated in the first 12 h of treatment, followed by a significant downregulation; *ClZFP86* was markedly upregulated during the first 24 h, followed by a sharp and significant decrease in expression.

Under the salt stress conditions, the expression patterns of the selected *CIZFP* gene family members exhibited polymorphic and dynamic fluctuations. *CIZFP14*, *CIZFP18*, *CIZFP19*, *CIZFP48*, *CIZFP52*, *CIZFP69*, and *CIZFP86* displayed distinct expression trends during the first 48 h of salt treatment, yet all of them reached the peak of upregulation at 72 h post-treatment, suggesting a collective role in long-term salt stress adaptation. Notably, *CIZFP26* showed extremely significant downregulation relative to the control at all time points under salt stress. In contrast, *ClZFP63* was strongly and continuously upregulated throughout salt treatment, highlighting its key role in salt stress tolerance.

## 4. Discussion

In recent years, the large-scale identification and analysis of gene families has emerged as a central focus of functional genomics research. To date, numerous relevant studies have been conducted in model plants, major crops, fruit crops, and other plant species. Watermelon (*Citrullus lanatus*), a globally important Cucurbitaceae horticultural crop, has been widely studied for gene family identification, including the *MYB*, *TPS*, *Aux/IAA*, *GRAS*, *TALE* and *PMEI* gene families [30,31,32,33,34,35]. However, despite the extensive investigations into various gene families in watermelon, the identification of the C2H2-ZFP family has not been reported yet. C2H2-ZFPs represent one of the largest and most conserved transcription factor families in plants, playing pivotal regulatory roles in multiple biological processes including organ morphogenesis, growth and development, and abiotic stress responses. In this study, we systematically identified 96 *ClZFP* family members in the watermelon genome and conducted a comprehensive analysis of their physicochemical properties, chromosomal localization, evolutionary relationships, gene structure, conserved motifs, tissue-specific expression patterns, and expression responses to drought, low temperature, and NaCl stress. These 96 ClZFP proteins showed marked diversity in physicochemical properties, with amino acid lengths spanning 131 aa to 1561 aa, indicative of profound functional differentiation among the members. Most of the ClZFP proteins were predicted to be unstable, a common feature of plant transcription factors that facilitates the dynamic regulation of target gene expression in response to internal and external signals, while the few stable members (*ClZFP23*, *ClZFP60*, *ClZFP82*) may serve as core regulators for basic cellular physiological processes. The subcellular localization prediction revealed most of the ClZFP proteins to be nuclear-localized, consistent with the canonical role of C2H2-ZFPs as transcription factors that bind specific DNA sequences and modulate gene transcription, and minor localization variations may suggest non-canonical functions of individual members in other cellular compartments. A phylogenetic analysis clustered the ClZFP proteins into six clades, with Clade III containing the largest number of members (39), and a subsequent conserved motif analysis revealed that all the members with abundant motifs were clustered in Clade III, indicating a strong correlation between the evolutionary classification of *ClZFP* genes and their structural characteristics. A gene structure and conserved motif analysis further revealed the structural conservation and divergence of the *ClZFP* gene family. The number of exons in the *ClZFP* genes ranged from 1 to 14, with 54 members containing only a single exon, suggesting that most of the *ClZFP* genes have a simple gene structure.

C2H2-ZFPs perform key functions in the development of various organs and structures throughout plant growth and development. The tissue-specific expression patterns of 12 selected *ClZFP* genes were investigated, which revealed their potential functional divergence during watermelon growth and development. In tomato, the overexpression of *ZAT1* (C2H2-ZFP) promotes the elongation of primary roots [36,37]. In watermelon, *ClZFP48* and *ClZFP69* were highly expressed in the roots, whereas *ClZFP36* was markedly repressed. These expression patterns suggest that these *ClZFP* genes may contribute to root development and root system establishment. In apple, *MdZAT10* promotes leaf senescence, and homologous genes in Arabidopsis have similar functions [38,39]. *ClZFP69* showed strong leaf-specific expression, suggesting a specialized role in leaf development or leaf-localized stress responses. C2H2-ZFPs are also known to regulate floral organ identity and development [40]. *ClZFP86* was specifically abundant in male flowers, and both *ClZFP86* and *ClZFP26* were highly expressed in female flowers. These observations indicate that *ClZFP* genes contribute to reproductive development and floral morphogenesis in watermelon.

In addition to their roles in plant development regulation, some C2H2-ZFPs are induced under abiotic stress, binding to the promoters of critical genes in different signaling pathways to regulate plant stress responses via modulation of the transcription levels of corresponding target genes [41,42]. C2H2-ZFPs play an active role in positively regulating drought, low-temperature and NaCl stress resistance in plants [14,15,16,17,43,44,45]. In agreement with these studies, *ClZFP52* was continuously induced by drought, supporting its role as a positive regulator of drought tolerance. Under cold stress, *ClZFP19*, *ClZFP36*, and *ClZFP63* were strongly upregulated, implying important functions in cold acclimation pathways. Notably, *ClZFP63* was persistently induced under salt stress, identifying it as a key candidate positive regulator of salt tolerance. Furthermore, *ClZFP19*, *ClZFP26*, *ClZFP36*, and *ClZFP72* were consistently repressed under drought conditions, suggesting they may act as negative regulators or participate in growth–stress trade-offs. Under salt stress, most of the *ClZFP* genes exhibited dynamic expression fluctuations and reached the peak of upregulation at 72 h post-treatment, which indicated that 72 h is a critical time point for the watermelon salt stress response, and the delayed upregulation of these genes may be associated with the activation of long-term salt tolerance mechanisms. Several of the *ClZFP* family members showed altered expression levels under 4 °C (6 h) treatment, according to previous transcriptome data in our laboratory [46]. The differences in the watermelon varieties used for the stress treatments resulted in divergent expression patterns of ClZFPs under identical stress conditions, further confirming the functional diversity of C2H2-ZFPs in watermelon. Given their broad and specific stress responsiveness, the *ClZFP* genes hold considerable potential for breeding watermelons with enhanced multiple stress tolerance. Despite these findings, several limitations should be acknowledged. First, this study is solely based on bioinformatic prediction and expression profiling, without in vivo functional validation such as transgenic overexpression, gene editing, or protein–DNA interaction assays. Second, the precise regulatory pathways involving ClZFPs—including their upstream regulators, direct target genes, and interactions with ABA, ROS, or CBF signaling modules—remain unresolved. Third, the phenotypic contributions of these genes to stress resistance in whole plants remain untested. In future research, the functional verification of these candidate genes and the dissection of their regulatory networks will help to clarify the molecular mechanisms of *ClZFP* genes in watermelon growth and stress adaptation and provide theoretical support and technical means for the cultivation of new high-quality and stress-resistant watermelon varieties.

## 5. Conclusions

C2H2-type zinc finger proteins (C2H2-ZFPs) are essential transcription factors governing plant growth, development, and abiotic stress tolerance, yet the family has not been systematically characterized in watermelon to date. In this study, we identified 96 *ClZFP* genes and analyzed their phylogenetic relationships, structural features, tissue-specific expression, and transcriptional responses to drought, cold, and salt stress, illustrating their functional diversification in development and stress adaptation. Notably, specific *ClZFP* members (e.g., *ClZFP52*, *ClZFP63*) exhibited distinct positive or negative regulatory roles under different abiotic stresses, while their tissue-specific expression further indicated their involvement in root, leaf and floral development. This work not only fills the research gap of the C2H2-ZFP family in watermelon but also provides important gene resources and a theoretical foundation for enhancing stress tolerance in cucurbit crops. The stress-responsive *ClZFP* genes identified in this study represent valuable candidate targets for molecular breeding programs aimed at cultivating high-yield and stress-resistant watermelon varieties.

## Figures and Tables

**Table 1:** Information of 96 ClZFP gene homologs in watermelon.

Gene Name	Gene ID	Protein Length (aa)	Molecular Weight (kDa)	pI	GRAVY	The Instability Index (II)	Aliphatic Index	Asp + Glu	Arg + Lys	Subcellular Location(s)
*ClZFP1*	*Cla97C01G004250*	281	31.95397	8.33	−0.902	57.37	62.17	41	44	Nuc.
*ClZFP2*	*Cla97C01G006090*	219	23.25366	6.13	−0.568	53.79	54.93	20	17	Nuc.
*ClZFP3*	*Cla97C01G007170*	387	43.33523	8.58	−0.645	68.72	69.87	46	52	Nuc.
*ClZFP4*	*Cla97C01G012770*	367	41.68777	8.73	−0.712	48.69	69.07	33	40	Nuc.
*ClZFP5*	*Cla97C01G018120*	373	41.62594	5.65	−0.495	44.28	63.08	54	43	Nuc.
*ClZFP6*	*Cla97C01G018790*	511	53.90265	8.63	−0.512	60.23	63.66	47	54	Nuc.
*ClZFP7*	*Cla97C01G020390*	215	22.98697	4.86	−1.004	62.36	45.44	38	25	Cyt.; Ext.; Nuc.
*ClZFP8*	*Cla97C01G022180*	355	39.70302	8.26	−0.526	59.48	72.25	47	51	Ext.; Nuc.
*ClZFP9*	*Cla97C01G025680*	373	40.02366	8.24	−0.329	59.29	72.63	28	30	Nuc.
*ClZFP10*	*Cla97C01G026200*	314	36.24835	8.5	−0.853	57.35	52.48	42	50	Cyt.; Nuc.
*ClZFP11*	*Cla97C02G028740*	438	48.63578	9.04	−0.629	46.96	59.02	35	48	Nuc.
*ClZFP12*	*Cla97C02G030100*	284	31.53544	7.62	−0.586	74.03	66.02	35	36	Cyt.
*ClZFP13*	*Cla97C02G033550*	207	22.91982	8.4	−0.65	45.25	61.88	20	22	Nuc.
*ClZFP14*	*Cla97C02G033740*	244	26.19504	8.46	−0.678	62.79	57.25	22	25	Nuc.
*ClZFP15*	*Cla97C02G036120*	171	19.10671	9.43	−0.699	70.55	62.81	15	21	Nuc.
*ClZFP16*	*Cla97C02G036320*	433	47.55877	9	−0.577	45.16	60.85	37	50	Nuc.
*ClZFP17*	*Cla97C02G045680*	371	41.16322	8.76	−0.252	62.19	85.2	40	47	Nuc.
*ClZFP18*	*Cla97C02G046380*	156	16.9733	7.66	−0.398	54.3	72.44	17	18	Nuc.
*ClZFP19*	*Cla97C02G047690*	299	33.14416	6.6	−0.616	60.31	72.78	37	34	Nuc.
*ClZFP20*	*Cla97C02G047930*	460	49.22612	4.75	−1.288	50	44.37	107	71	Nuc.
*ClZFP21*	*Cla97C02G049440*	364	40.84292	6.38	−0.848	54.64	53.35	38	32	Nuc.
*ClZFP22*	*Cla97C03G052140*	192	21.14765	5.7	−0.657	69.39	69.06	24	19	Nuc.
*ClZFP23*	*Cla97C03G052910*	137	14.97217	9.11	−0.5	29.56	62.7	13	19	Nuc.
*ClZFP24*	*Cla97C03G053270*	419	46.19312	9.27	−0.613	42.85	63.87	33	46	Nuc.
*ClZFP25*	*Cla97C03G054490*	1561	174.87058	8.81	−0.729	55.52	64.37	203	227	Nuc.
*ClZFP26*	*Cla97C03G060550*	423	46.25392	9.32	−0.499	56.12	67.14	37	59	Cyt.
*ClZFP27*	*Cla97C03G060870*	277	31.22495	7.93	−0.837	62.12	50.04	30	32	Nuc.
*ClZFP28*	*Cla97C03G067400*	427	46.53521	9.1	−0.489	50.58	66.49	38	51	Nuc.
*ClZFP29*	*Cla97C04G069010*	357	39.2988	6.36	−0.743	61.32	54.93	39	33	Nuc.
*ClZFP30*	*Cla97C04G070400*	290	32.00044	8.77	−0.566	43.97	58.1	31	41	Ext.
*ClZFP31*	*Cla97C04G074610*	242	27.48591	8.93	−0.808	67.41	60.04	29	35	Cyt.; Nuc.
*ClZFP32*	*Cla97C04G075160*	160	17.86052	9.27	−0.551	63.17	71.38	16	22	Nuc.
*ClZFP33*	*Cla97C04G075590*	848	93.58299	9.13	−0.698	48.59	60.09	68	94	Nuc.
*ClZFP34*	*Cla97C04G077240*	407	46.48243	9.08	−0.884	54.67	66.14	46	60	Nuc.
*ClZFP35*	*Cla97C04G078840*	515	58.0397	8.74	−0.89	54.68	51.57	67	76	Nuc.
*ClZFP36*	*Cla97C04G079800*	451	49.91297	8.85	−0.789	58.56	61.26	53	63	Nuc.
*ClZFP37*	*Cla97C05G080200*	378	42.6029	5.59	−1.032	75.51	53.2	69	53	Nuc.
*ClZFP38*	*Cla97C05G080210*	589	66.24553	6.3	−0.971	52.62	58.35	89	75	CM.; Nuc.
*ClZFP39*	*Cla97C05G085430*	160	18.41626	6.11	−1.066	49.91	48.81	21	17	Nuc.
*ClZFP40*	*Cla97C05G085440*	192	21.36093	8.94	−0.649	40.66	66.15	20	24	Cyt.; Nuc.
*ClZFP41*	*Cla97C05G090240*	709	79.58054	6.21	−0.705	47.17	75.95	105	96	Nuc.
*ClZFP42*	*Cla97C05G092750*	204	23.20212	6.15	−0.798	77.83	63.63	28	24	Nuc.
*ClZFP43*	*Cla97C05G096080*	438	47.32023	9.39	−0.357	40.89	73.9	39	61	Ext.
*ClZFP44*	*Cla97C05G097460*	206	22.65397	6.6	−0.743	64.03	61.65	24	23	Nuc.
*ClZFP45*	*Cla97C05G099210*	411	45.72971	8.98	−0.742	43.47	58.44	35	46	Nuc.
*ClZFP46*	*Cla97C05G099660*	422	45.87016	9.51	−0.488	59.77	69.6	37	61	Nuc.
*ClZFP47*	*Cla97C05G101770*	417	47.84202	7.16	−0.684	49.99	70.1	66	66	Chl.; Cyt.; Mit.; Nuc.
*ClZFP48*	*Cla97C05G102250*	262	27.8158	7.21	−0.572	63.84	64.92	24	24	Nuc.
*ClZFP49*	*Cla97C05G103060*	339	37.75012	6.57	−0.776	63.02	64.19	38	35	Nuc.
*ClZFP50*	*Cla97C05G104390*	223	23.47929	7.72	−0.366	44.1	59.19	18	19	Nuc.
*ClZFP51*	*Cla97C05G105800*	450	51.06584	9.25	−1.092	52.86	51.36	64	80	Nuc.
*ClZFP52*	*Cla97C05G107100*	284	31.71685	8.41	−0.456	62.14	63.2	24	28	Nuc.
*ClZFP53*	*Cla97C05G109160*	464	52.73399	8.67	−0.687	52.36	58.47	59	70	Cyt.; Nuc.
*ClZFP54*	*Cla97C06G114240*	136	14.60151	8.97	−0.309	45.5	67.5	8	12	Nuc.
*ClZFP55*	*Cla97C06G116410*	166	18.40637	5.77	−0.724	74.79	60.54	18	15	Ext.
*ClZFP56*	*Cla97C06G125210*	588	67.36224	5.35	−1.188	49.18	52.45	122	100	Cyt.; Er.; Nuc.
*ClZFP57*	*Cla97C06G116390*	164	18.63898	9.11	−0.922	60.16	59.51	23	28	Nuc.
*ClZFP58*	*Cla97C06G125360*	492	55.2082	8.96	−0.826	62.84	62.84	64	76	Nuc.
*ClZFP59*	*Cla97C07G129150*	184	21.22031	7.69	−1.145	49.98	49.29	21	22	Cyt.; Nuc.
*ClZFP60*	*Cla97C07G129160*	200	22.32428	5.96	−0.999	39.9	46.8	24	18	Cyt.; Nuc.
*ClZFP61*	*Cla97C07G131490*	172	18.39384	9.27	−0.43	57.64	68.08	12	19	Nuc.
*ClZFP62*	*Cla97C07G131880*	131	15.08619	9.67	−0.7	59.23	62.6	10	17	Nuc.
*ClZFP63*	*Cla97C07G134720*	397	44.80245	5.2	−1.022	55.31	52.52	74	53	Nuc.
*ClZFP64*	*Cla97C07G138870*	266	28.62793	8.35	−0.569	45.98	67.52	24	26	Nuc.
*ClZFP65*	*Cla97C08G150370*	1473	164.99889	5.93	−0.622	57.49	69.93	209	181	Nuc.
*ClZFP66*	*Cla97C08G150630*	229	25.21563	9.31	−0.548	59.46	67.47	20	32	Nuc.
*ClZFP67*	*Cla97C08G157050*	215	23.65346	9.37	−0.672	63.76	59.95	17	23	Nuc.
*ClZFP68*	*Cla97C08G158810*	459	47.21437	9.12	−0.174	60.57	70.46	29	41	Nuc.
*ClZFP69*	*Cla97C08G160110*	234	25.34045	8.98	−0.612	80.36	57.65	21	26	Nuc.
*ClZFP70*	*Cla97C09G162690*	526	56.97357	8.91	−0.645	52.86	57.78	41	52	Nuc.
*ClZFP71*	*Cla97C09G165510*	248	27.27036	5.89	−0.52	53.23	71.61	27	22	Nuc.
*ClZFP72*	*Cla97C09G166500*	309	34.43893	8.34	−0.638	56.05	55.63	26	30	Nuc.
*ClZFP73*	*Cla97C09G167760*	198	21.52205	9.94	−0.943	55.86	49.85	19	33	Nuc.
*ClZFP74*	*Cla97C09G168750*	254	28.83679	9.85	−0.889	54.78	57.99	20	44	Nuc.
*ClZFP75*	*Cla97C09G170040*	508	55.28856	8.69	−0.648	43.15	60.16	40	47	Nuc.
*ClZFP76*	*Cla97C09G173130*	508	56.2001	5.82	−0.584	47.53	66.85	67	55	Nuc.
*ClZFP77*	*Cla97C09G174860*	259	28.48544	5.89	−0.643	55.68	58.42	24	18	Nuc.
*ClZFP78*	*Cla97C09G183550*	166	18.82308	9.14	−0.598	54.24	57.11	14	19	Nuc.
*ClZFP79*	*Cla97C10G184740*	240	25.59814	5.79	−0.922	59.45	58.17	33	29	Nuc.
*ClZFP80*	*Cla97C10G186340*	196	21.68272	5.65	−0.67	58.7	59.29	23	16	Nuc.
*ClZFP81*	*Cla97C10G187180*	265	28.89624	7	−0.768	72.93	58.19	39	39	Nuc.
*ClZFP82*	*Cla97C10G187640*	367	41.88385	9.12	−0.593	34.7	78.86	54	64	Nuc.
*ClZFP83*	*Cla97C10G188350*	248	27.84808	5.16	−0.881	72.38	69.6	47	34	Nuc.
*ClZFP84*	*Cla97C10G188360*	234	25.38154	9.27	−0.644	49.62	61.32	25	33	Nuc.
*ClZFP85*	*Cla97C10G190310*	435	46.85144	9.53	−0.407	48.54	72.64	38	60	Ext.
*ClZFP86*	*Cla97C10G197410*	194	21.15816	8.88	−0.611	49.44	50.82	23	33	Ext.
*ClZFP87*	*Cla97C10G200100*	353	40.83314	6.29	−1.171	74	42.86	50	41	Ext.; Nuc.
*ClZFP88*	*Cla97C10G202520*	314	34.31458	4.79	−1.11	45.83	52.23	66	45	Nuc.
*ClZFP89*	*Cla97C10G203050*	244	26.80316	7.6	−0.596	67.86	66.84	26	27	Nuc.
*ClZFP90*	*Cla97C10G203560*	493	53.5049	9.34	−0.574	52.88	67.77	39	54	Nuc.
*ClZFP91*	*Cla97C10G204610*	250	28.38481	5.47	−1.108	72.28	50.32	42	30	Nuc.
*ClZFP92*	*Cla97C10G205190*	332	38.54631	9.47	−0.849	54.67	54.58	34	61	Nuc.
*ClZFP93*	*Cla97C11G215620*	141	15.91001	8.49	−0.672	72.15	62.98	13	15	Ext.
*ClZFP94*	*Cla97C11G218870*	311	33.48309	6.41	−0.585	53.59	67.78	33	30	Nuc.
*ClZFP95*	*Cla97C11G219330*	371	42.5523	6.18	−1.151	60.78	49.95	64	55	Cyt.; Nuc.
*ClZFP96*	*Cla97C00G000030*	401	42.56333	9.14	−0.481	58.26	65.34	35	48	Nuc.

Nuc. = nucleus; Cyt. = cytoplasm; Ext. = extracellular; Chl. = chloroplast; Mit. = mitochondrion; CM. = cell membrane; Er. = cndoplasmic reticulum.

## Data Availability

The original contributions presented in this study are included in the article/Supplementary Materials. Further inquiries can be directed to the corresponding authors.

## Supplementary Materials

The following supporting information can be downloaded at: https://www.mdpi.com/article/10.3390/genes17050504/s1, Table S1: The basic information of 96 C2H2-ZFP genes; Table S2: Real-time fluorescent quantitative PCR primers; Table S3: Collinear relationships of C2H2-ZFP family members between watermelon and Arabidopsis.
